# Impact of IFN lambda 3/4 single nucleotide polymorphisms on the cytomegalovirus reactivation in autologous stem cell transplant patients

**DOI:** 10.1371/journal.pone.0200221

**Published:** 2018-07-23

**Authors:** Ombretta Annibali, Livia Piccioni, Valeria Tomarchio, Erika Circhetta, Chiara Sarlo, Luca Franceschini, Maria Cantonetti, Emanuela Rizzo, Silvia Angeletti, Maria Cristina Tirindelli, Carolina Scagnolari, Maura Statzu, Giuseppe Avvisati, Elisabetta Riva

**Affiliations:** 1 Unit of Hematology, Stem Cell Transplantation, Campus Bio-Medico University, Rome, Italy; 2 Laboratory of Virology, Campus Bio-Medico University, Rome, Italy; 3 Hematology and Stem Cell Transplant Unit, Tor Vergata University, Rome, Italy; 4 Laboratory of Pathology and Microbiology, Campus Bio-Medico University, Rome, Italy; 5 Transfusion Medicine and Cellular Therapy, Campus Bio-Medico University, Rome, Italy; 6 Department of Molecular Medicine, Laboratory of Virology, “Sapienza” University, Rome, Italy; National Institute of Health, ITALY

## Abstract

Cytomegalovirus (CMV) infection represents one of the main cause mortality after Stem Cell Transplantation. Recently, a protective effect of the T allele of rs12979860 IL28B Single Nucleotide Polymorphisms (SNPs) against CMV infection in the allogenic stem cell transplantation was suggested. We investigate whether the rs12979860 IL28B SNP and the relative rs368234815 (IFNλ4) genotype may affect the incidence of active CMV infection in Autologous stem cell transplantation (Auto-SCT) setting. The study included 99 patients who underwent to Auto-SCT. IL28 and IFNΔ4 SNPs were correlated with CMV reactivation along with other clinical and treatment parameters. CMV reactivation by CMV DNAemia was evaluated once a week until day 100 from Auto-SCT. CMV reactivation was documented in 50% (TT-ΔG/ΔG), 35% (CC-TT/TT) and 29.2% (CT-TT/ΔG) of the patients respectively. No differences in CMV copies number were recorded at reactivation between different IL28/IFNλ4 genotypes. The analysis of patients older than 60 years showed a significantly higher incidence of active CMV infection in the TT-ΔG/ΔG (83%) population with respect to CC-TT/TT (21%) and CT-TT/ΔG (40%) patients. Our data suggest a negative role of TT-ΔG/ΔG genotype in the CMV reactivation in Auto-SCT. The exposure to rituximab and the pre-infusion presence of anti CMV IgG also significantly influenced CMV reactivation.

## Introduction

Cytomegalovirus (CMV) infection represents one of the main cause of morbidity and mortality after stem cell transplantation (SCT) because the deep immunosuppression contributes significantly to the loss of CMV-specific adaptive immune control [1]. Thus, the role of the innate immunity to control CMV replication is magnified in transplant setting. Type III interferons IFNs), including IFNλ1, IFNλ2 and IFNλ3 also known as IL29, IL28A and IL28B respectively, are thought to display antiviral and immunomodulatory properties *in vivo*, which may partially overlap those exerted by type I IFNs. [2–3] Type I and Type III IFNs both generate an antiviral state by triggering the JAK-STAT pathway, ultimately upregulating the expression of IFN stimulated genes. [2–3]. The rs12979860 IL28B single nucleotide polymorphism (SNP) is well known to influence the spontaneous and treatment-induced clearance in HCV infection [4–9]. However, attempts to identify a functional mechanism linked to IL-28B SNPs have failed to yield consistent results. Recently, Prokunina-Olsson et al. [10] pinpoint a dinucleotide rs368234815 frameshift variant (previously named ss469415590 TT/ΔG) that is upstream of and in the same orientation as IL28B able to generate a novel IFNλ protein, called IFNλ4. Individuals who carry the homozygosis for the minor ΔG allele of the ss469415590 variant ΔG/ΔG genotypes) can produce IFNλ4, whereas the presence of the major TT allele leads to a frameshift in exon 1 and disrupts the IFNλ4 open reading frame. Linkage disequilibrium (LD) between IFNλ4-ΔG, which creates IFNλ4 protein, and the (unfavorable) IFNλ3 rs12979860-T allele was demonstrated to be very high among Caucasians (>0.9) [10]. This means that these variants are always inherited together making sometime difficult to determine which one is more strongly associated with the outcome and, therefore, more likely to be causal. Thus, the “so called” unfavorable rs12979860 TT genotype co-migrates with the ΔG/ΔG IFNλ4 type and is itself strictly associated with the production of the IFNλ4 protein. Despite there is strong evidence that genotypes for rs12979860 and IFNλ4- TT/ΔG may play a role in other infections [11–16], their relevance in CMV reactivation following stem cell transplantation is still debated. Several lines of evidence suggest that IL28B/IFNλ4 SNPs might play a role in the control of CMV infection in Allo-SCT and solid organ cells transplant recipients [1,17,18]; however, to date no data are available in autologous stem cells transplantation (Auto- SCT) setting. The current study was aimed at investigating factors that may be involved in CMV reactivation and whether the rs12979860 IFNλ3 polymorphism and the relative rs368234815 IFNλ4 genotype may have any effect on the incidence rate and outcome of active CMV infection in Auto-SCT.

## Methods

This study was approved by Campus Bio-Medico ethical committee (n.02.15TS.COMETCBM).All patients included in this study provided written, informed consent.

### Patients

Starting from October 2014, 99 consecutive patients who underwent Auto-SCT for hematological malignancies, were recruited at the Haematologic Departments of Campus Bio-Medico and Tor Vergata University Hospitals. They were screened for complete laboratory assessment, total-body computerized tomography scan (CT scan), virological pre-transplantation assessment and for IL28B and IFNλ4 SNPs genotyping. Virological and bacterial infections were recorded and evaluated up to 100 days following Auto-SCT. Antiviral prophylaxis with Acyclovir was administered immediately before the infusion and maintained for 1 year. CMV reactivation by CMV DNAemia was evaluated once a week until day 100 from Auto-SCT. Pre-emptive therapy with i.v. Ganciclovir (5mg/kg/12 hr) or i.v. Foscarnet (60 mg/kg/12hr) was initiated either upon CMV DNAemia threshold level of 1000 IU/mL and/ or in presence of organ disease (hepatic, pulmonary gastro-enteric) and discontinued after two consecutive negative results. During episodes of active CMV infection, CMV surveillance was performed twice a week, when possible.

### Screening for hepatitis viruses

Antigen/antibodies screening for hepatitis viruses (A,B,C) was performed by Chemio Luminescent Immuno Assay (CLIA) using a sandwich test for antigen (HBsAg, HBeAg) or antibodies (anti HCV, Anti HBsAg, Anti HBeAg, Anti HBcAg total and IgM) (Centaur, Siemens Healthcare, Italy). HBV DNA and HCV RNA were performed by using COBAS Ampliprep TaqMan48 according to the Manufacturer’s instructions (Roche, Italy).

Antibodies against CMV (IgG and IgM) were evaluate by Diasorin CLIA essay on LIAISON XL Instruments (Diasorin, Italy).

### Cytomegalovirus DNAemia

Cytomegalovirus DNA was extracted from plasma samples by versant KPCR Molecular System SP station and quantified by Real Time PCR assay in KPCR Molecular System AD (Siemens Healthcare, Italy). The detection limit of the test is 200 IU/mL. Active CMV infection was defined as the detection of CMV DNA in more than one plasma specimens. The duration of a given episode of CMV DNAemia was considered to comprise the interval between the day of first detection of CMV DNA in plasma and that of the first negative PCR results.

### Single nucleotide polymorphism analysis

Genomic DNA was extracted from whole blood by using QIAamp DNA Mini Kit according to the manufacturer's instruction. The Rs12979860 IL28B SNP (C/T) genotype was determined by Real Time PCR followed by Melting analysis (PCR Light Mix Kit IL28B -TIB MOLBIOL-ROCHE, Italy) on DNA obtained as previously described. Genotyping for IFNλ4-TT/ΔG (rs368234815) was performed with custom TaqMan allelic discrimination genotyping assays on the LightCycler® 480 System (Roche, Basel, Switzerland). For quality control, blinded duplicate specimens were included in the panel. Primers and probes were selected according to previously report [10].

### Statistical methods

Patients’ characteristics were summarized by means of frequency (n) and percentage (%) for categorical variables or by means of median and range for continuous variables. Differences among groups (IL28, CMV reactivation) were evaluated in univariate analysis by means of non-parametric tests (Chi-Squared and Fisher Exact test in case of categorical variables, Mann-Whitney and Kruskal-Wallis test in case of continuous variables) and logistic regression model in multivariate analysis. Boxplots were used to show CMV copies at reactivation among IL28B polymorphism levels. Survival CMV reactivation was estimated using the Kaplan-Meier Product Limit estimator and the differences among groups were evaluated by means of Log-Rank test. All tests were 2-sided, accepting P<0.05 as indicating a statistically significant difference and confidence intervals were calculated at 95% level. All analyses were performed using the R software (R Foundation for Statistical Computing, Vienna, Austria. ISBN 3-900051-07-0).

## Results

### Patients

Clinical and demographic data of the 99 patients are summarized in Table 1.

**Table 1:** Baseline patients’ characteristics.

Number of patients	99
Median age (min-max)	56 (18–66)
Gender (M/F)	61/38
Disease	
MM	77
NHL	82
Conditioning regimen	
MEL 200	68
MEL 140	3
MEL 100	6
FEAM	12
BEAM	10
Median CD4+ infused value	
CD4+/Kg (min-max)	5x10^6^/Kg (2–11.2)
Engraftment (Days)	
PMN >500/mmc	11 (3–83)
Ptls >20000/mmc	13 (9–41)

MM: Multiple Myeloma; NHL: Non Hodgkin Lymphoma; MEL: Melphalan (200 mg, 140 mg or 100 mg); BEAM: Carmustine, Etoposide, Cytarabine and Melphalan FEAM: Fotemustine plus Etoposide, Cytarabine and Melphalan; PML: Polymorphonucleate; Plts: Platelets.

Multiple Myeloma (MM) patients were conditioned with Melphalan (200 mg, 140 mg or 100 mg) while subjects with Non Hodgkin Lymphoma received BEAM (#10) and Fotemustine plus Etoposide, Cytarabine and Melphalan (FEAM #12). Before Auto-SCT, all patients received viral screening as indicated in Table 2.

**Table 2:** Pre-transplant viral screening.

Viral screening pre-Auto SCT*	(neg/pos)
HBsAg (neg/pos)	97/2
AntiHBs Ab	91/8
AntiHBc Ab	85/14
HBV DNA	98/1
AntiHCV Ab	99/0
HCV RNA	99/0
AntiCMV IgG (pos/neg)	77/22

*Auto-Stem Cells transplantation

After Auto-SCT and until day 100 after the infusion, all patients were monitored for infective complications. CMV reactivation was observed in 34/99 (34%) patients, bacterial infections were detected in 32/99 patients (32%), while fungal infections were recorded in 1/99 (1%) patients. Because all 34 patients with active CMV DNAemia were positive for anti CMV IgG, we refer to a reactivation of infection rather than a primary infection.

Among the patients with CMV reactivation, 13 had NHL and 21 MM, representing 59% and 21% of the total NHL and MM patients respectively.

### Polymorphism study

Genetic screening for IL-28B rs12979860 (C/T) and IFNλ4 rs368234815 (TT/ΔG) was performed in all patients. As shown in Table 3, CC–TT/TT genotype was recorded in 46% of patients while CT–TT/ΔG and TT -ΔG/ΔG was detected in 41% and 12% of the subjects, respectively. Our analysis confirmed the strong Linkage Disequilibrium (LD) between these two polymorphisms and the same T/ΔG minor allele frequency (MAF) of the Caucasian general population (MAF 0.3).

**Table 3:** Genetic screening and polymorphisms of IL28B rs12979860 and IFNλ4 rs368234815.

	IL28B		
IFNλ4	CC	CT	TT
TT/TT	46(46%)	0	0
TT/ΔG	0	41(41%)	0
ΔG/ΔG	0	0	12(12%)

### Cytomegalovirus reactivation

Cytomegalovirus reactivation was found in 34/99 patients (34%). Fifty percent (6/12) of the TT-ΔG/ΔG showed a CMV reactivation whereas, among patients bearing CC-TT/TT and CT-TT/ΔG, 35% (16/46) and 29% (12/41), respectively, experienced CMV reactivation (P = ns). As showed in Table 4, when the analysis was restricted in the setting of patients aged >60 years (n = 30), CMV reactivation was significantly higher in patients with TT-ΔG/ΔG genotype (83%) rather than in CT-TT/ΔG (40%) or in CC-TT/TT patients (21%) (P<0.05).

**Table 4:** CMV reactivation and IL28B polymorphisms in patients aged >60 years.

IL28B/IFNλ4	CC-TT/TT	CT-TT/ΔG	TT-ΔG/ΔG	P-value
total	14	10	6	
CMV reactivation (%)				
No	11 (78.6)	6 (60.0)	1 (16.7)	
yes	3 (21.4)	4 (40.0)	5 (83.3)	P<0.05

**Fig 1:**
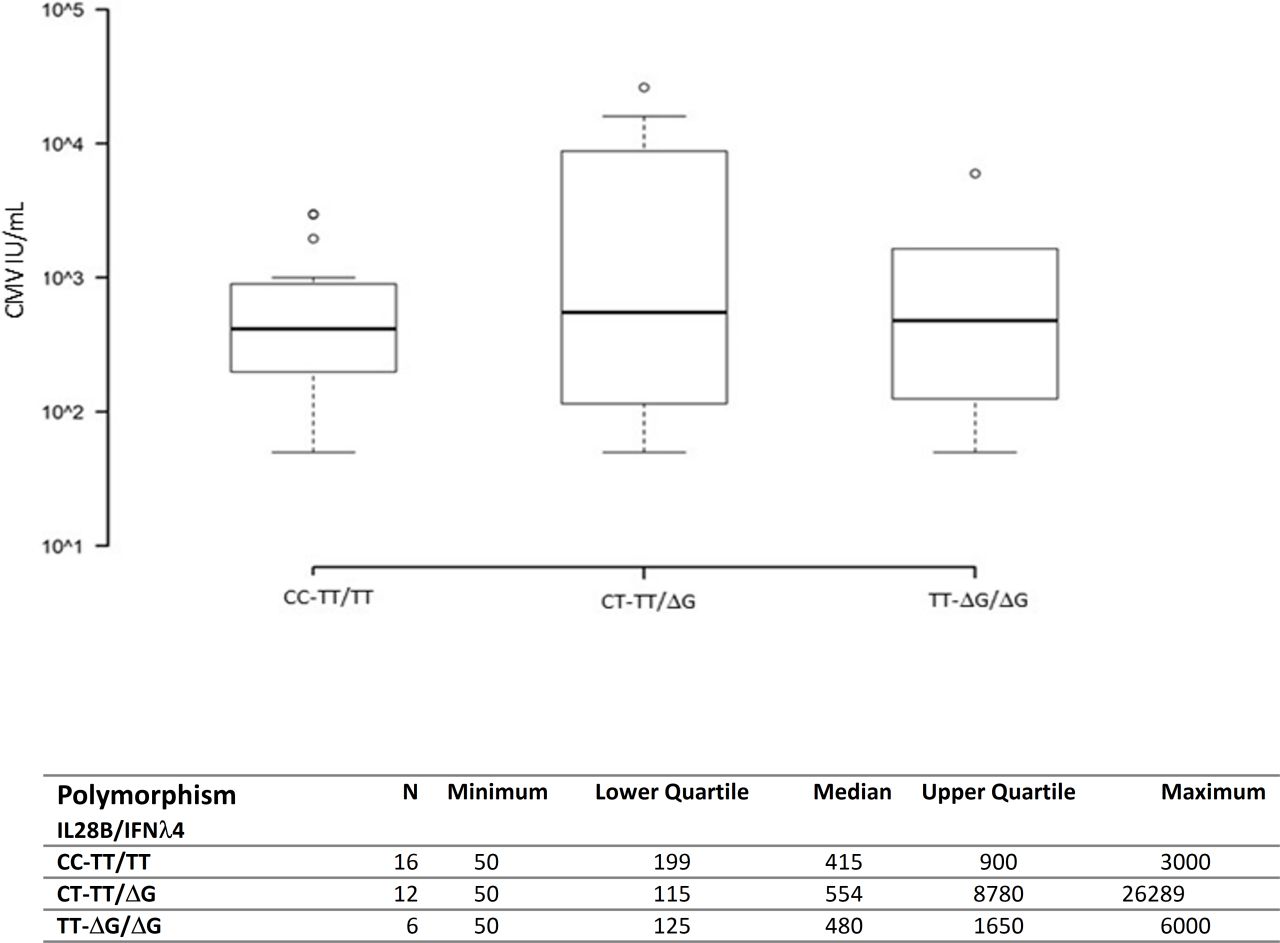
CMV DNAemia at reactivation distributed between different IL28B/IFNλ4 genotypes.

The analysis of median CMV DNA copies number at the reactivation was not significantly correlated with a specific IL28B/IFNλ4 genotype, although higher levels of CMV DNAemia were recorded in the heterozygote CT-TT/ΔG carriers when compared to the other groups (Fig 1).

No difference was observed between Gangyclovir or Foscarnet as for treatment response to antiviral treatment and disease recurrence by genetic polymorphisms.

### Univariate and multivariate analysis

Univariate analysis highlighted that the presence of a diagnosis of NHL (P = 0.05), exposure to immunotherapy with Rituximab (P = 0.0046), conditioning regimen with FEAM (P = 0.0058), IgG positivity for CMV (P = 0.03) and the presence of bacterial infection (P = 0.045) were variables that significantly influenced CMV reactivation.

**Fig 2:**
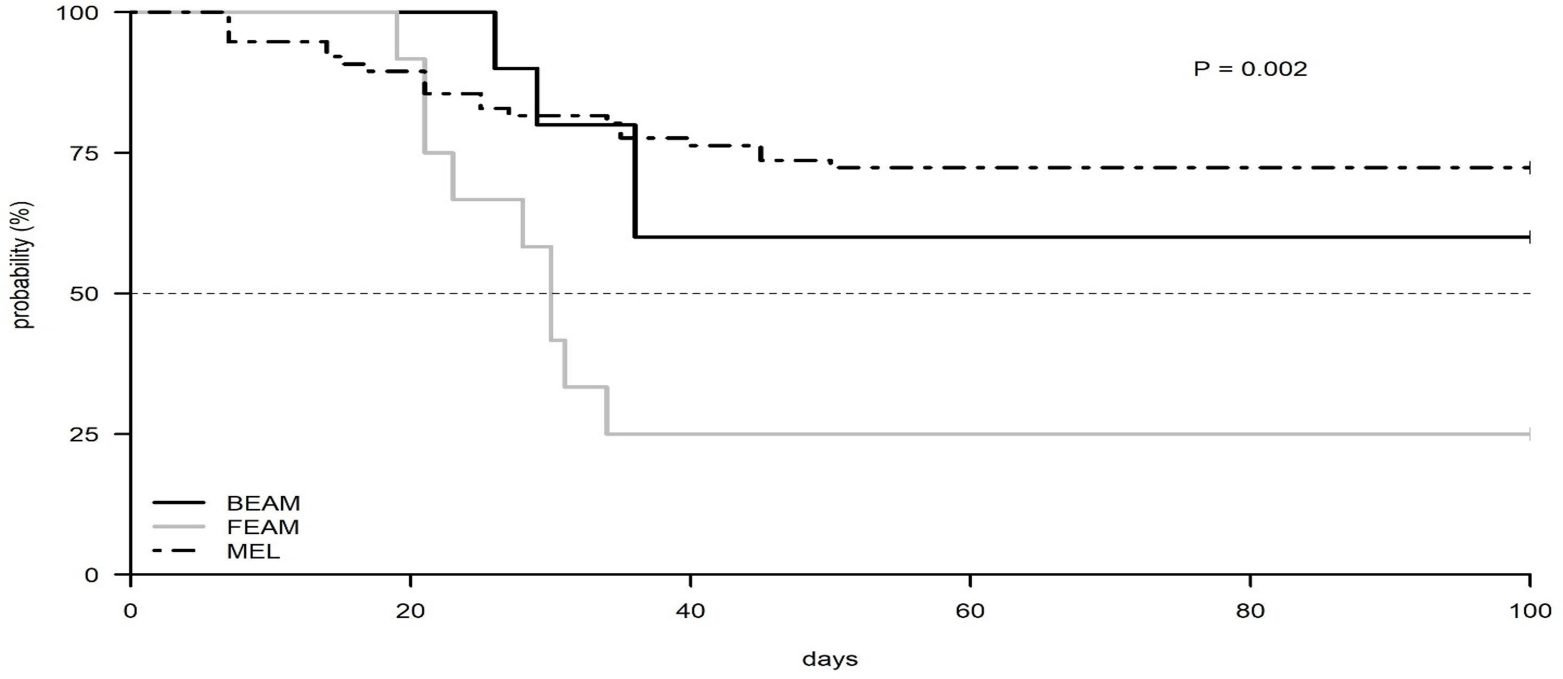
Kaplan Meier plot of CMV reactivation according to the type of conditioning chemotherapy regimen (BEAM vs. FEAM vs. MEL).

**Fig 3:**
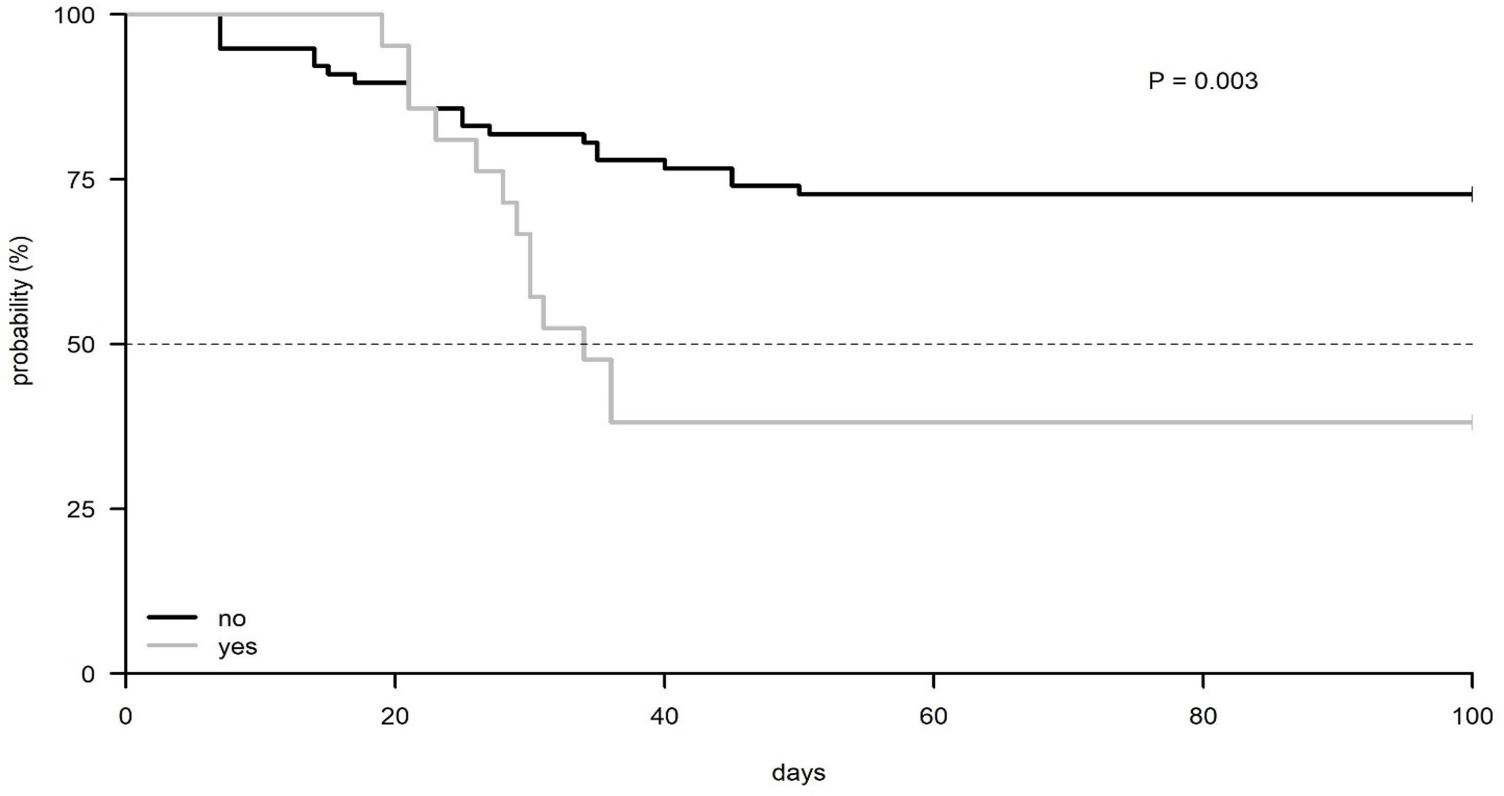
Kaplan Meier plot of CMV reactivation according to Rituximab infusion received (yes vs. no).

The monitoring of the CMV reactivation during 100 days post-transplantation by type of conditioning regimen received, showed that patients receiving FEAM had an earlier (median 30 days) CMV reactivation (P = 0.0018) compared to those receiving BEAM (median not reached, 65% reactivation) (Fig 2). Importantly, patients with MM who received high-dose MEL as conditioning showed a lower incidence of reactivation compared to both BEAM and FEAM regimen. Moreover, CMV reactivation was significantly higher within 60 days after Auto-SCT in patients treated with Rituximab when compared to the group that did not (61% vs 27.3% P<0.0034) (Fig 3).

Multivariate analysis showed that only exposure with rituximab (P = 0.007) and the presence of CMV IgG significantly influenced CMV reactivation (P = 0.04).

## Discussion

In recent years, gene-polymorphism studies have increased considerably. Scientific evidence showed a very close link between the presence of defined polymorphisms in genes and their involvement in the regulation and activation of innate and adaptive immune responses against persistent viral infection like HCV or CMV. In this contest, the analysis of many polymorphism, such as CCR5 (*chemokine receptor 5*), MCP-1 (*monocyte chemoattractant protein 1*), IL10 (*interleukin 10*), DC-SIGN (*dendritic cell-specific molecule-3-grabbing non integrin*), highlighted a correlation between certain of these SNPs and an increased risk of CMV infection in patients who underwent Allo-SCT [19,20]. The rs12979860 (C/T) single nucleotide polymorphism in IL28B gene region is well known to influence the spontaneous and treatment-induced clearance in HCV infection [4–9]. In particular, the presence of the C allele and the CC homozygosis has shown to guide positively the virus clearance. A strong Linkage disequilibrium (LD) between IFNλ4-ΔG allele and the (unfavorable) IL28B rs12979860 T allele was demonstrated to be very high in the Caucasians (>0.9) [10], meaning that variants are always inherited together. The presence of ΔG allele is associated with the production of IFNλ4 protein and with impaired HCV clearance. Due to the strong LD between the two SNPs, the rs12979860 T of IL-28B allele may act as a marker for IFNλ4 protein production. Recently, a key role of the rs12979860 IL-28B SNP in the reactivation of some Herpesvirus and CMV infection in the Allo-SCT setting was also suggested.

For example, the retrospective study of Bravo et al. [17], showed a trend towards a lower incidence of active CMV infection in the TT genotype stem cell donors (even not statistically significant). In the same time, the Egli’s group confirmed that solid organ transplant recipients with a minor-allele genotype (TT) showed a trend to less CMV replication (CC vs TT, 52% vs 0%; *P* = 0.089) [1], suggesting a protective effect of the rs12979860 T allele against CMV infection and reactivation in immunocompromised patients.

By contrast, data from Manuel et al. [18] showed that solid transplanted patients, carrying the IFNλ4 ΔG/ΔG genotype, have a higher but statistically not significant cumulative incidence of CMV replication, compared with TT/TT or TT/ΔG carriers. In this study, the association becomes statistically significant among patients followed by a preemptive approach, especially in those receiving an organ from a seropositive donor but not among those who received antiviral prophylaxis [18].

The discordant results obtained by different groups in different transplantation setting (Allo-SCT or solid organ), make very difficult to really understanding the clinical relevance of such SNPs on CMV reactivation making further prospective studies necessary. Moreover, no data, regarding the correlation between IL28B/IFNλ4 and CMV reactivation in Auto-SCT setting, are available until now.

Our data suggest a significantly higher risk of CMV reactivation in NHL rather than in MM and, among NHL patients, an increased risk of reactivation in subjects who received FEAM as conditioning chemotherapy.

We also found that rituximab immunotherapy increased significantly the risk of CMV reactivation respect to MM who did not receive Rituximab, probably due to its immunosuppressive effect. The analysis of other risk factors showed no correlation between viral reactivation and disease-stage at transplantation or with the presence of antibody against HBV, HCV and HIV or previous exposition to proteasome inhibitors as also demonstrated by Marchesi and coworkers [21].

Interestingly, we noticed that the onset of bacterial infection was significantly related to CMV reactivation in univariate but not into multivariate analysis, thus considering a bacterial infection as a driver for CMV reactivation only in presence of other factors (i.e. immunosuppressive therapy).

The analysis of the correlation between IFNλ3 rs12979869 CT-TT/ΔG and IFNλ4 SNPs and CMV reactivation showed that TT-ΔG/ΔG subjects reactivated more frequently (50%) than CT-TT/ΔG (29%) or CC-TT/TT (35%) patients.

Considering only patients aged ≥60 years, CMV reactivation was significantly higher in TT-ΔG/ΔG genotype (83%) when compared to CT-TT/ΔG (40%) and CC-TT/TT (21%) (P = 0.03). The achievement of statistical significance in this specific subgroup of our population might be explained considering that the T/ΔG MAF rises from 0.3 to 0.4, rendering the minor allele T/ΔG sufficiently represented.

Our results agree with what observed in HCV infection where, carrying TT-ΔG/ΔG genotype negatively influenced the clearance of infection but, significantly contrast with the evidence of Bravo and Egli in Allo-SCT and solid transplantation setting, respectively [17,1]. On the other hand, our data remark those obtained by Manuel et al. [18] who demonstrated a highest cumulative incidence of CMV replication in ΔG/ΔG patients followed by a pre-emptive approach in solid transplantation.

Even the results of these studies are hard to compare because they have been led using patients managed in different setting of transplantation, the main differences may be explained considering that in the Auto-SCT setting the donor/recipients are the same, leading to consider only one type of immune system. By contrast, in Allo-SCT (but also in the solid transplantation setting) we must take into account both the donor’s and the recipient’s immune system, making the framework of interpretation more complex.

The exact mechanism by which these SNPs exert their activity is not well established. Although the expression of IFNλ ligands seems to be modulate in both transcription factors binding and methylation sites of the promoter [22–24], the impact of such SNPs on IFNλ expression is still debated. Some studies suggested that the presence of the “unfavorable” rs12979860 T allele is associated with both reduced IFNλ3 expression and with higher and prolonged ISG expression, especially in HCV infection [6–8, 25–31]. By contrast, the P70S aminoacid substitution in IFNλ4 protein has been shown to decrease IFN-related antiviral activity by the reduction of ISG expression levels [31]. Beside the relevance in controlling the innate immune signaling, an important impact on adaptive immune function, such as virus-induced B cells proliferation, antibody production and cytokines dysfunction, has also been proposed in acute infections [32– 33]. Thus, the dual antiviral (by innate immunity) and immunomodulatory role of IL28B/IFNλ4 can result in multiple possible interactions leading to a difficult and intriguing puzzle to resolve.

In conclusion, our study, which is the first performed in Auto-SCT setting, supports the idea that the TT-ΔG/ΔG genotype is negatively involved in CMV reactivation and suggests that TT-ΔG/ΔG patients aged ≥60 years are at increased risk of CMV reactivation as well as those with NHL or conditioned with FEAM.

The relatively low number of patients may limit our results and thus prospective and longer studies in Auto-SCT transplant setting are needed to confirm and validate such findings.

## Supporting information

S1 Table Patients’ Dataset supporting the analysis. (XLSX)
